# Analysis of risk factors for depression and anxiety in women with polycystic ovary syndrome

**DOI:** 10.3389/fgwh.2025.1520641

**Published:** 2025-03-03

**Authors:** Yanting Yang, Lin Liu, Ning Hu, Huyan Huo, Xin Yang, Fang Wang

**Affiliations:** ^1^ Department of Obstetrics and Gynecology, Second Clinical Medical College, Lanzhou University, Lanzhou, China; ^2^ Department of Reproductive Medicine, Second Clinical Medical College, Lanzhou University, Lanzhou, China; ^3^ Department of Reproductive Medicine, Second Hospital, Lanzhou University, Lanzhou, China

**Keywords:** polycystic ovary syndrome, depression, anxiety, sleep disorders, mental health

## Abstract

**Background:**

Polycystic ovary syndrome (PCOS) is one of the most common reproductive endocrine disorders among women of reproductive age, often accompanied by a series of symptoms such as hirsutism, hair loss, menstrual disorders and obesity, resulting in an increasing risk of depression and anxiety in such patients.

**Methods:**

A total of 413 patients in the Reproductive Medicine Center of the Second Hospital of Lanzhou University from June 2021 to June 2023 were enrolled. We collected sociodemographic information and lifestyle-related factors using a structured questionnaire. Patient Health Questionnaire (PHQ-9) and Generalized Anxiety Disorder Scale (GAD-7) were used to evaluate the psychological status of the subjects. Sleep-related variables were assessed using the Pittsburgh Sleep Quality Index (PSQI), and metabolic measures were collected from patients' medical records.

**Results:**

Compared with the control group, PCOS patients were younger, the average age was (27.39 ± 3.48) years old, and the BMI value was higher, the difference was statistically significant (*p* < 0.05). The proportions of depression and anxiety in PCOS patients were 47.7% and 39.9%, respectively. In PCOS patients with depressive anxiety symptoms, the proportions of mild, moderate, moderately severe and severe depression were 31.6%, 12.4%, 1.6% and 2.1%, respectively. The proportions of mild, moderate, moderately severe and severe anxiety were 30.6%, 6.2%, 1.0% and 2.1%, respectively. Depression was significantly associated with serum free triiodothyronine (FT3) OR (95% CI) = 3.33 (1.30–8.55), sleep duration 4.99 (1.45–17.23) and daytime dysfunction 8.24 (3.53–19.22). Anxiety was significantly associated with daytime dysfunction OR (95% CI) = 3.45 (1.78–6.70). No association was found between mental health and other metabolic characteristics in PCOS patients (*p* > 0.05).

**Conclusion:**

According to the results of the current study, a high proportion of women with PCOS have mental health disorders, and there is a significant correlation between mental health disorders and sleep conditions.

## 1 Introduction

Polycystic ovary syndrome (PCOS) is a multifaceted endocrine disorder affecting women of reproductive age, and it is responsible for almost 80% of cases of anovulatory infertility (1, 2). The condition is typically characterized by menstrual disorders (oligomenorrhea or amenorrhea), infertility, hyperandrogenemia, and polycystic changes in the ovaries, which impact women's reproductive, metabolic, and mental and emotional health (3, 4). Clinical research has revealed that, alongside reproductive disorders, patients with PCOS also exhibit endocrine, glucose, and lipid metabolism abnormalities as well as mental and psychological symptoms (5). Anxiety, depression, and other adverse emotions are the most prevalent mental and psychological issues in PCOS patients. Surveys indicate that the incidence of anxiety and depression in PCOS patients is 3–4 times higher than that of the general population (6, 7). Concerns about the risk of anxiety and depression in women with PCOS are increasing (8, 9).

The pathophysiological mechanism underlying the higher prevalence of anxiety or depression in women with PCOS remains unclear and has not been definitively linked to a specific risk factor. However, studies have shown that factors such as obesity, insulin resistance and elevated androgen levels may be involved in the pathogenesis of PCOS (10). A cross-sectional study found that the occurrence of depression in PCOS patients is correlated with their quality of life, and the incidence of depression in PCOS patients is increased, and the quality of life is significantly decreased (11).Farrell et al. found that PCOS is associated with obesity, infertility, hirsutism and acne, and these factors may contribute to the development of depression (12). Some systematic reviews have also indicated that the prevalence of anxiety symptoms is significantly higher in women with PCOS compared to those in the normal control group, and the presence of anxiety symptoms may be associated with hirsutism, obesity, or infertility in patients with PCOS (13). The median prevalence of depressive symptoms in women with PCOS is 36.6%, and the prevalence of anxiety is as high as 76.7%; and women with PCOS have a higher risk of anxiety than women without PCOS (14). The literature review shows that there is good evidence that adult women with PCOS are at increased risk of depression and anxiety symptoms (15). Clinical studies have shown that sleep disturbances occur more frequently in women with PCOS than in normal controls, and also have shown that the proportion of sleep disorders in PCOS patients is 16.14% (16). Sleep disturbances may be related to anxiety and depression to some extent, but the specific impact of sleep disorders on the psychological health of women with PCOS has not been studied so far (17). Currently, the etiology of the elevated prevalence of depression and anxiety in women with PCOS remains unclear, thus necessitating further research.

The aim of this study was to investigate the correlation between metabolic indicators, sleep status and anxiety and depression in women with PCOS.

## 2 Materials and methods

### 2.1 Study population

**Figure 1:**
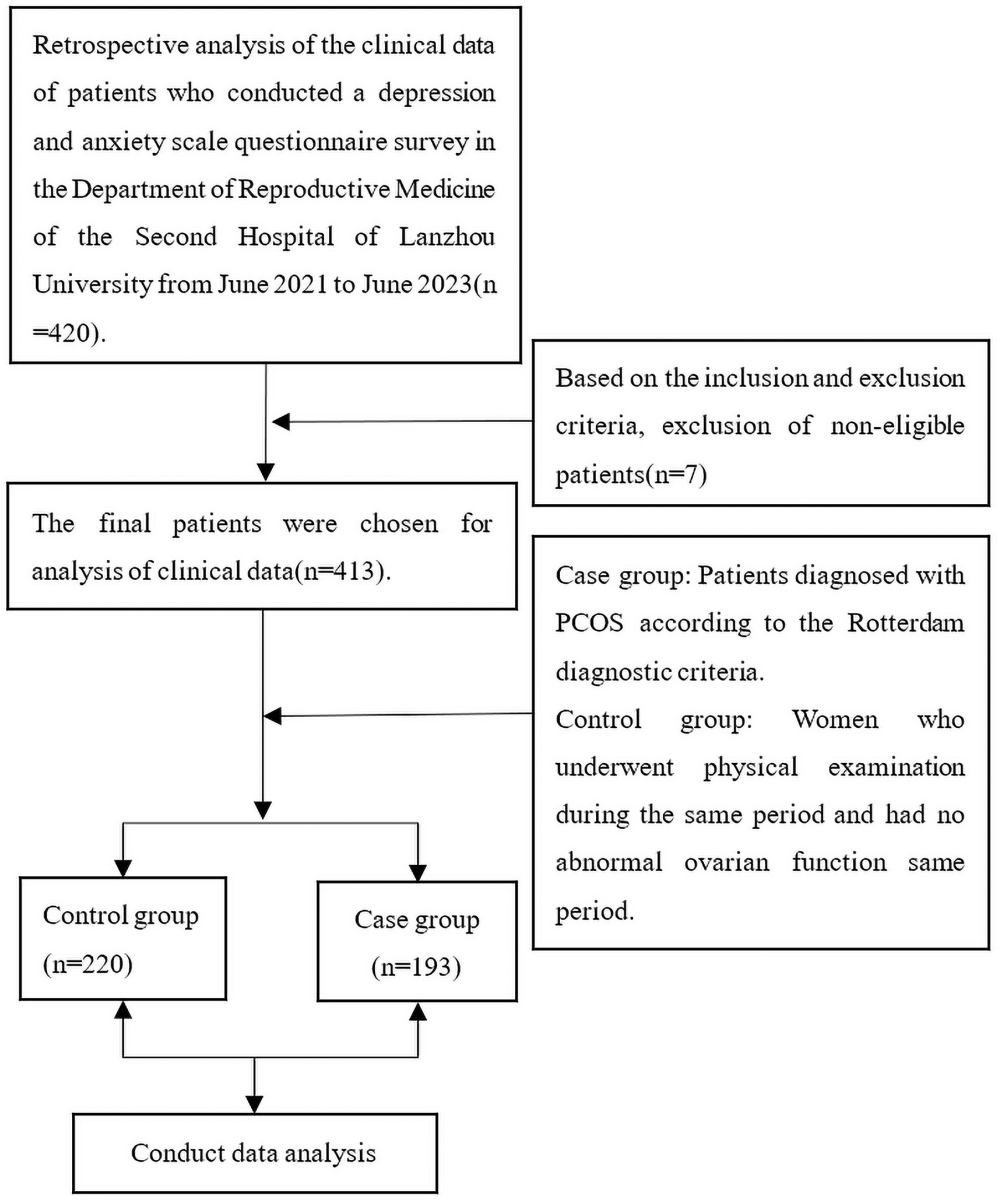
Study flow chart.

The data of this study were collected from patients who visited the Reproductive Medicine Center of the Second Hospital of Lanzhou University from June 2021 to June 2023. Patients included in the study were between 20 and 40 years of age. Patients were excluded from the study if one of the following conditions was met: (1) the participant was unable to complete the questionnaire independently; (2) taking hormones or drugs affecting hormone secretion in the past three months; (3) History of congenital adrenal hyperplasia, Cushing's syndrome, or thyroid dysfunction; (4) ovarian or adrenal tumors causing ovulation disorder or abnormal androgen secretion; (5) They are in the lactation period; (6) previous history of malignant tumor; The case group consisted of patients diagnosed with PCOS, while the control group comprised patients without PCOS. To be diagnosed with PCOS according to the Rotterdam criteria (18), women had to have at least two of the following symptoms: (1) oligoovulation or anovulation; (2) clinical and/or biochemical hyperandrogenism, including acne, hypertrichosis, and seborrheic alopecia; (3) Ultrasound showed ovarian polycystic changes(Ultrasound showed that there were more than 12 follicles with a diameter of 2–9 mm in one or both ovaries, and/or the ovarian volume was more than 10 cm^3^). The control group consisted of women who underwent a healthy physical examination and had no abnormal ovarian function during the same period. The study flow chart is shown in Figure 1.

### 2.2 Sample size estimation

Based on the literature, the estimated incidence of PCOS ranges from 10%–13%. The report indicates an expected prevalence of 13% (19). Two-sided tests were conducted with a precision of 5% and confidence levels at 95%, using the sample size formula:

$$n=(Z_{1-}\alpha /2/\delta {)}^{2}\times P\times (1-P).$$

The calculated sample size was 174, where *Z*_1−*α*_/2 represents the statistic for the confidence level, *δ* is the exact level, and *P* is the anticipated prevalence (20). A minimum enrollment of 209 patients was necessary to accommodate for a potential 20% missing data. This estimate is conservative due to an expected higher prevalence in women of reproductive age. Ultimately, this study included a sufficient sample size with a total of 413 hospitalized patients.

### 2.3 Demographic characteristics and scale assessment

After signing the consent form, height and weight were measured, and body mass index (BMI) was calculated as weight (kg)/height (m)^2^. The questionnaire included demographic characteristics (such as age, ethnicity, education level, smoking, alcohol consumption and second-hand smoke exposure, family history of hypertension and diabetes), anxiety, depression, and sleep status. The anxiety scale and depression scale were completed by professional doctors to guide patients during the visit to assess whether patients had anxiety and depression symptoms.

Sleep related variables were assessed by Pittsburgh Sleep Quality Index (PSQI). PSQI scale was used to evaluate the sleep quality of the subjects in the past month, including 19 items in 7 factors, including sleep quality, sleep latency, sleep duration, sleep efficiency, sleep disorders, sleep drug use and daytime function. Each factor was scored according to 3 points, and the score of each factor component was accumulated into the total score of PSQI scale. The total score ranges from 0–21 points.

Depressive symptoms at baseline were assessed using the 9-item Patient Health Questionnaire (PHQ-9) (21). PHQ-9 scores with major depression diagnoses from validated diagnostic interviews. Items are scored on a four-point Likert scale from 0 (“not at all”) to 3 (“nearly every day”). Item scores are summed to a total ranging from 0–27 whereby higher values indicate higher depressive symptoms. Score: <5 points without depression, 5–9 is mild depression; 10–14 is moderate depression; ≥15 is considered severe depression.

Anxiety symptoms at baseline were assessed using the Generalized Anxiety Disorder 7-item Scale (GAD-7) (22). Items are scored on a four-point Likert scale from 0 (“not at all”) to 3 (“nearly every day”). Item scores are summed to a total ranging from 0–21 whereby higher values indicate higher anxiety symptoms. Score: <5 points without Anxiety, 5–9 is mild Anxiety; 10–13 is moderate Anxiety; ≥14 is considered severe Anxiety.

### 2.4 Laboratory data sources

All researchers collected fasting venous blood from the outpatient department of the Second Hospital of Lanzhou University. Follicle stimulating hormone (FSH), luteinizing hormone(LH), serum free T3 (FT3), serum free T4 (FT4), thyroid stimulating hormone (TSH), homocysteine (HCY), 25-OH-vitamin D (25-OH-VitD), fasting blood glucose (FBG), fasting insulin (FINS), fasting C-peptide (FCP), 2 h blood glucose (PBG), cholesterol (CHO), triglyceride(TG), high density lipoprotein (HDL), low density lipoprotein (LDL), aspartate aminotransferase (AST), alanine aminotransferase (ALT), uric acid (UA), creatinine (Cr) and urea (BUN) was completed by the Laboratory of the Second Hospital of Lanzhou University. All are measured by the automatic biochemical detector (ACLTOP750LAS) of Werfen Medical Device Co., Ltd. in the United States, and all operations are carried out in strict accordance with the instructions.

Insulin resistance index (HOMA-IR) = FBG*FINS/22.5, The unit of FBG is mmol/L, and the unit of FINS is mU/L, and the coefficient 22.5 is the correction factor.

### 2.5 Data analyses

Statistical analyses were performed with the use of the SPSS statistical package, version 25.0. Continuous variables with a normal distribution were expressed as mean ± SD, and continuous variables without a normal distribution were expressed as interquartile ranges (p25, p75). For categorical variables, we expressed them as numbers and percentages. t test and non-parametric test were used for measurement data, and *χ*^2^ test was used for count data. Logistic regression was used to analyze the relationship between mental health (anxiety, depression) and sleep disorders and biochemical indicators. Results were expressed as odds ratio (OR) and 95% confidence interval (CI). Two-sided *p* < 0.05 was considered to statistical significance.

## 3 Results

### 3.1 The baseline characteristics of the two groups were compared

The demographic characteristics of the healthy and PCOS groups are presented in Table 1. A total of 413 women were enrolled in this study, comprising 220 non-PCOS women as the control group and 193 PCOS women as the case group. The results presented in the table indicate that there were no statistically significant differences observed between the control and case groups regarding smoking habits, exposure to second-hand smoke, family history of hypertension, and family history of diabetes (*p* > 0.05). However, notable disparities were found in terms of age, BMI, ethnicity, and education level between the two groups (*p* < 0.05). Specifically to the control group, patients with PCOS exhibited younger age and higher BMI values. There are also significant differences between the two groups in terms of education level and nationality (*p* < 0.05) (Table 1).

**Table 1:** Demographic features of individuals with PCOS compared to those with normal physical examination results.

Variable	Control Group	Case Group	Z/*χ*2	*p-*Value
	(*n* = 220)	(*n* = 193)		
Age(year)	33 (30, 37)	27 (25,29)	Z = −12.97	<0.001
Age categories(*n*%)			χ2 = 155.68	<0.001
<30 year	40 (18.2)	147 (76.2)		
30–35 year	87 (39.5)	41 (21.2)		
≥35 year	93 (42.3)	5 (2.6)		
BMI(Kg/m^2^)	22 (20,24)	24 (21,28)	*Z* = −6.60	<0.001
BMI categories(*n*%)			χ2 = 44.43	<0.001
<25 Kg/m^2^	184 (83.6)	103 (53.4)		
≥25 Kg/m^2^	36 (16.4)	90 (46.6)		
Nation(*n*%)			χ2 = 15.91	<0.001
Han	211 (95.9)	168 (87)		
Hui ethnic group	9 (4.1)	13 (6.7)		
Other nationalities	0 (0.0)	12 (6.2)		
Education(*n*%)			χ2 = 54.74	<0.001
Junior high	16 (7.3)	54 (28.0)		
Senior high	9 (4.1)	30 (15.5)		
University	195 (88.6)	109 (56.5)		
Smoking(*n*%)			χ2 = 0.01	0.93
No	214 (97.3)	188 (97.4)		
Yes	6 (2.7)	5 (2.6)		
Second-hand smoke exposure(*n*%)			χ2 = 0.58	0.87
No	82 (37.3)	79 (40.9)		
Yes	138 (62.7)	114 (59.1)		
Family hypertension(*n*%)			χ2 = 2.88	0.45
No	134 (60.9)	133 (68.9)		
Yes	86 (39.1)	60 (31.1)		
Family diabetes(*n*%)			χ2 = 0.03	0.87
No	186 (84.5)	162(83.9)		
Yes	34(15.5)	31(16.1)		

Bolded portions indicate categorical variables, and comparisons between groups were made using chi-square tests.

### 3.2 Depression and anxiety were compared between the two groups

The depression and anxiety levels of the two groups are presented in Table 2 for comparative analysis. Significant differences were observed between the two groups in terms of total score, severity, symptoms, and overall anxiety score. Furthermore, a higher proportion of patients with PCOS exhibited comorbidities of anxiety and depression when compared to the general population.

**Table 2:** Depression and anxiety were compared between the two groups.

Variable	Control Group	Case Group	Z/χ2	*p-*Value
	(*n* = 220)	(*n* = 193)		
Total depression score	4 (2,8)	7 (2,11)	Z = −3.89	<0.001
Depression(*n*%)			χ2 = 4.16	0.041
No	137 (62.3)	101 (52.3)		
Yes	83 (37.7)	92 (47.7)		
Depression symptom(*n*%)			χ2 = 9.71	0.046
Mild	54 (24.5)	61 (31.6)		
Moderate	14 (6.4)	24 (12.4)		
Moderately severe	7 (3.2)	3 (1.6)		
Severe	8 (3.6)	4 (2.1)		
Total anxiety score Anxiety	2 (0,6)	4 (2,6)	Z = −4.78	<0.001
Anxiety(*n*%)			χ2 = 1.49	0.22
No	145 (65.9)	116 (60.1)		
Yes	75 (34.1)	77 (39.9)		
Anxiety symptom(*n*%)			χ2 = 6.98	0.14
Mild	49 (22.3)	59 (30.6)		
Moderate	12 (5.5)	12 (6.2)		
Moderately severe	9 (4.1)	2 (1.0)		
Severe	5(2.3)	4(2.1)		

Bolded portions indicate categorical variables, and comparisons between groups were made using chi-square tests.

### 3.3 Comparison of the general condition of depression or not in patients with PCOS

A comparison of the demographic characteristics of the PCOS population with respect to depression is shown in Table 3. There were no statistically significant differences in age, BMI, education, smoking, secondhand smoke exposure, family history of hypertension, and family history of diabetes between PCOS patients with and without depression (*p* > 0.05) (Table 3). The two groups were statistically different with respect to ethnicity and having fertility problems (*p <* 0.05) (Table 3). Moreover, depression was more common in Han women (Han nationality is the main nationality in China) with PCOS, and the proportion of fertility problems was higher (Table 3).

**Table 3:** Comparison of general baseline data between women with and without depression in PCOS(*N* = 193).

Variable	Total (*n* = 193)	Depression-	Depression+	Z/χ2	*p*-value
		(*n* = 101)	(*n* = 92)		
Age(year)		27.41 ± 3.31	27.37 ± 3.67	Z = 0.072	0.942
Age categories(*n*%)				χ2 = 2.88	0.237
<30 year	147 (76.2)	76 (75.2)	71 (77.2)		
30–35 year	41 (21.2)	24 (23.8)	17 (18.5)		
≥35 year	5 (2.6)	1 (1.0)	4 (4.3)		
BMI(Kg/m^2^)		24.70 ± 4.36	25.80 ± 4.84	Z = −1.67	0.096
BMI categories(*n*%)				χ2 = 2.17	0.141
<25 Kg/m^2^	103 (53.4)	59 (58.4)	44 (47.8)		
≥25 Kg/m^2^	90 (46.6)	42 (41.6)	48 (52.2)		
Nation(*n*%)				χ2 = 8.03	0.018
Han	168 (87.0)	83 (82.2)	85 (92.4)		
Hui ethnic group	13 (6.7)	7 (6.9)	6 (6.5)		
Other nationalities	12 (6.2)	11 (10.9)	1 (1.1)		
Education(*n*%)				χ2 = 5.44	0.066
Junior high	54 (28.0)	34 (33.7)	20 (21.7)		
Senior high	30 (15.5)	11 (10.9)	19 (20.7)		
University	109 (56.5)	56 (55.4)	53 (57.6)		
Smoking(*n*%)				χ2 = 0.121	1.000
No	188 (97.4)	98 (97.0)	90 (97.8)		
Yes	5 (2.6)	3 (3.0)	2 (2.2)		
Second-hand smoke exposure(*n*%)				χ2 = 0.155	0.694
No	79 (40.9)	40 (39.6)	39 (42.4)		
Yes	114 (59.1)	61 (60.4)	53 (57.6)		
Family hypertension(*n*%)				χ2 = 1.120	0.290
No	133 (68.9)	73 (72.3)	60 (65.2)		
Yes	60 (31.1)	28 (27.7)	32 (34.8)		
Family diabetes(*n*%)				χ2 = 0.008	0.930
No	162 (83.9)	85 (84.2)	77 (83.7)		
Yes	31 (16.1)	16 (15.8)	15 (16.3)		
Family fertility problem(*n*%)				χ2 = 5.78	0.016
No	179 (92.7)	98(97.0)	81(88.0)		
Yes	14(7.3)	3(3.0)	11(12.0)		

Bolded portions indicate categorical variables, and comparisons between groups were made using chi-square tests.

### 3.4 Comparison of the general condition of anxiety or not in patients with PCOS

A comparison of anxious and non-anxious demographic characteristics in the PCOS population is shown in Table 4. There were no significant differences in age, BMI, education level, smoking, second-hand smoke exposure, family history of hypertension, and family history of diabetes between PCOS patients with and without anxiety (*p* > 0.05) (Table 4). There were significant differences between the two groups with respect to ethnicity (*p* < 0.05) (Table 4). In addition, anxiety was more common in Han women with PCOS (Table 4). However, the limited ethnic diversity in this study, primarily consisting of Han population, diminishes the comparative significance of the observed differences.

**Table 4:** Comparison of general baseline data between women with and without anxiety in PCOS(*N* = 193).

Variable	Total	Anxiety-	Anxiety+	Z/χ2	*p*-value
	(*n* = 193)	(*n* = 116)	(*n* = 77)		
Age(year)		27.45 ± 3.67	27.30 ± 3.20	Z = 0.292	0.771
Age categories(*n*%)				χ2 = 1.497	0.473
<30 year	147 (76.2)	85 (73.3)	62 (80.5)		
30–35 year	41 (21.2)	28 (24.1)	13 (16.9)		
≥35 year	5 (2.6)	3 (2.6)	2 (2.6)		
BMI(Kg/m^2^)		25.24 ± 4.39	25.20 ± 4.96	Z = 0.057	0.954
BMI categories(*n*%)				χ2 = 0.734	0.392
<25 Kg/m^2^	103 (53.4)	59 (50.9)	44 (57.1)		
≥25 Kg/m^2^	90 (46.6)	57 (49.1)	33 (42.9)		
Nation(*n*%)				χ2 = 6.582	0.037
han	168 (87.0)	98 (84.5)	70 (90.9)		
Hui ethnic group	13 (6.7)	7 (6.0)	6 (7.8)		
Other nationalities	12 (6.2)	11 (9.5)	1 (1.3)		
Education(*n*%)				χ2 = 2.959	0.228
Junior high	54 (28.0)	37 (31.9)	17 (22.1)		
Senior high	30 (15.5)	15 (12.9)	15 (19.5)		
University	109 (56.5)	64 (55.2)	45 (58.4)		
Smoking(*n*%)				χ2 = 0.210	0.647
No	188 (97.4)	112 (96.6)	76 (98.7)		
Yes	5 (2.6)	4 (3.4)	1 (1.3)		
Second-hand smoke exposure(*n*%)				χ2 = 0.550	0.458
No	79 (40.9)	45 (38.8)	34 (44.2)		
Yes	114 (59.1)	71 (61.2)	43 (55.8)		
Family hypertension(*n*%)				χ2 = 1.664	0.197
No	133 (68.9)	84 (72.4)	49 (63.6)		
Yes	60 (31.1)	32 (27.6)	28 (36.4)		
Family diabetes(*n*%)				χ2 = 0.064	0.800
No	162 (83.9)	98 (84.5)	64 (83.1)		
Yes	31 (16.1)	18 (15.5)	13 (16.9)		
Family fertility problem(*n*%)				χ2 = 0.643	0.423
No	179 (92.7)	109(94.0)	70(90.9)		
Yes	14(7.3)	7(6.0)	7(9.1)		

Bolded portions indicate categorical variables, and comparisons between groups were made using chi-square tests.

### 3.5 Association of biochemical indicators with depression/anxiety

The relationship between biochemical indicators and anxiety/depression is shown in Table 5. There were no significant differences in hormone levels, blood lipids, FBG, FINS, HOMA-IR, FCP, thyroid function, liver enzyme, renal function, and homocysteine between the anxiety/depression group and the non-anxiety/depression group. However, in PCOS patients with/without depression, we found a significant difference in FT3 between the two groups (OR 3.33; 95% CI 1.3–8.55; *p* < 0.05) (Table 5). It indicates that thyroid function may have a certain effect on depression in women, and more research is needed to find out the specific effect. Because some patients did not undergo biochemical tests, the biochemical indicators of all patients could not be obtained. Partial missing values (missing values < 0.05) of non-biochemical results were filled by multiple imputation method.

**Table 5:** The biochemical indicators of anxiety/depression in PCOS patients were compared (univariate logistic regression).

Index	Depression+		Anxiety+	
	OR (95% CI)	*p*-Value	OR (95% CI)	*p*-Value
Age(year)	0.96 (0.84,1.09)	0.496	1 (0.88,1.14)	0.997
BMI(Kg/m^2^)	1.08 (0.94,1.24)	0.295	1.05 (0.92,1.21)	0.453
FSH(mIU/ml)	1.05 (0.67,1.62)	0.845	1 (0.66,1.5)	0.995
LH(mIU/ml)	0.94 (0.82,1.07)	0.346	0.98 (0.9,1.08)	0.697
FSH/LH	0.76 (0.19,2.98)	0.694	1.01 (0.88,1.17)	0.848
FT3 (pmol/L)	3.33 (1.30,8.55)	**0**.**012**	2.44 (0.93,6.36)	0.069
FT4 (pmol/L)	0.82 (0.64,1.04)	0.094	0.84 (0.66,1.08)	0.179
TSH(uIU/ml)	1.14 (0.94,1.39)	0.187	1.20 (0.98,1.46)	0.074
FBG(mmol/L)	1.19 (0.32,4.48)	0.796	0.64 (0.19,2.16)	0.469
FINS(mU/L)	0.95 (0.70,1.30)	0.763	0.91 (0.67,1.22)	0.509
HOMA-IR	1.03 (0.34,3.12)	0.963	1.38 (0.48,3.95)	0.554
FCP(ng/ml)	1.36 (0.34,5.51)	0.667	0.76 (0.19,3.00)	0.696
PBG(mmol/L)	0.92 (0.66,1.28)	0.628	1.00 (0.72,1.38)	0.992
CHO(mmol/L)	6.46 (0.85,49.04)	0.071	3.87 (0.47,31.89)	0.209
TG(mmol/L)	0.80 (0.38,1.68)	0.549	1.18 (0.56,2.51)	0.666
LDL(mmol/L)	0.16 (0.02,1.38)	0.095	0.20 (0.02,2.03)	0.173
HDL(mmol/L)	0.32 (0.04,2.65)	0.292	0.20 (0.02,2.18)	0.188
ALT(U/L)	0.96 (0.92,1.01)	0.114	0.99 (0.95,1.03)	0.579
AST(U/L)	1.07 (0.99,1.16)	0.083	1.05 (0.97,1.13)	0.229
AST/ALT	0.81 (0.44,1.47)	0.482	1.09 (0.63,1.88)	0.770
BUN(mmol/L)	0.29 (0.05,1.74)	0.175	0.39 (0.07,2.2)	0.283
Cr(*μ*mol/L)	1.14 (0.97,1.35)	0.118	1.02 (0.87,1.2)	0.780
UA(μmol/L)	1.00 (0.99,1.00)	0.206	1 (0.99,1.01)	0.710
HCY(μmol/L)	0.94(0.86,1.03)	0.152	1.07(0.99,1.17)	0.091

Bold numbers signify those with statistical significance.

### 3.6 Relationship between sleep and depression/anxiety

The associations between sleep factors and anxiety and depression are shown in Tables 6, 7. The results of univariate analysis showed that The OR (95%CI) values of depression and subjective sleep quality, sleep duration, sleep disorder, daytime dysfunction, total sleep score and sleep quality respectively were 2.08 (1.17–3.70), 4.94 (1.84–13.29), 2.68 (1.28–5.62),8.63 (3.94–18.93), 1.43 (1.21–1.70) and 4.58 (2.15 -9.76). The multivariate analysis still found statistically significant differences between depression and sleep duration as well as daytime dysfunction after taking into account all factors with significant statistical significance (Table 7). Similarly, the OR (95%CI) values of anxiety and subjective sleep quality, sleep duration, daytime dysfunction, total sleep score, and sleep quality assessment were 2.11 (1.18–3.78), 2.24 (1. 05–4.78), 3.94 (2. 11–7.39), 1.28 (1.09–1.50) and 2.45 (1.24–4.87). A statistically significant difference between anxiety and daytime dysfunction could still be seen by multivariate analysis of statistically significant factors.

**Table 6:** Single factor logistic regression analysis of sleep and anxiety/depression in PCOS patients (*N* = 193).

Variable	Depression+		Anxiety+	
	OR (95% CI)	*p*-Value	OR (95% CI)	*p*-Value
Subjective Sleep quality	2.08 (1.17, 3.70)	**0**.**013**	2.11 (1.18, 3.78)	**0**.**012**
Sleep latency	1.17 (0.86, 1.58)	0.325	1.22 (0.89, 1.66)	0.215
Sleep duration	4.94 (1.84, 13.29)	**0**.**002**	2.24 (1.05, 4.78)	**0**.**036**
Sleep efficiency				
>85%	1.0	NE	1.0	NE
≤85%	1.2 (0.79, 1.83)	0.404	0.91 (0.45, 1.83)	0.788
Sleep disturbance	2.68 (1.28, 5.62)	**0**.**009**	1.94 (0.98, 3.83)	0.056
Daytime dysfunction	8.63 (3.94, 18.93)	**<0**.**001**	3.94 (2.11, 7.39)	**<0**.**001**
Use of sleep medication				
No	1.0	NE	1.0	NE
Yes	NE	NE	NE	NE
Total score of PSQI	1.43 (1.21, 1.70)	**<0**.**001**	1.28 (1.09, 1.50)	**0**.**002**
Sleep quality assessment				
Good	1.0	NE	1.0	NE
No good	4.58 (2.15, 9.76)	**<0**.**001**	2.45 (1.24, 4.87)	**0**.**010**

NE, not available.

Bold numbers signify those with statistical significance.

**Table 7:** Multivariate logistic regression analysis of sleep and anxiety/depression in PCOS patients (*N* = 193).

Variable	Depression+		Anxiety+	
	ORs (95% CIs)	*p*-Value	ORs (95% CIs)	*p*-Value
Subjective sleep quality	1.68 (0.75, 3.76)	0.205	1.63 (0.79,3.37)	0.188
Sleep duration	4.99 (1.45, 17.23)	**0**.**011**	1.82 (0.72,4.59)	0.208
Sleep disturbulence	2.39 (0.82, 6.90)	0.109	1.61 (0.66,3.97)	0.297
Daytime dysfunction	8.24 (3.53, 19.22)	**<0**.**001**	3.45 (1.78,6.70)	**<0**.**001**
Total score of PSQI	0.82 (0.57, 1.19)	0.296	1.01 (0.74,1.37)	0.964
Sleep quality assessment				
Good	1.0	NE	1.0	NE
No good	2.16 (0.57, 8.21)	0.260	1.08 (0.33,3.56)	0.898

NE, not available.

Bold numbers signify those with statistical significance.

## 4 Discussion

With the evolution of the modern medical model, an increasing number of clinical studies have revealed that, in addition to reproductive and metabolic symptoms, PCOS patients exhibit a significantly higher incidence of anxiety and depression compared to healthy individuals (8, 10). Our study showed that approximately 47.7% of PCOS patients exhibited symptoms of depression, while 39.9% expressed symptoms of anxiety. The incidence of depression and anxiety was much higher than that of the normal control group (depression accounted for 37.7%, anxiety accounted for 31.1%), and the comparison between the two groups was statistically significant (*p* < 0.05). There is evidence that PCOS has many similarities with anxiety and depression in terms of pathological mechanism (23, 24). Their pathogenesis involves chronic inflammatory response, imbalance of neurotransmitters, disorder of endocrine system and abnormal reaction of immune system (25, 26). It may also be related to genetic factors, pressure of living environment, bad living habits and so on (27, 28). These similarities may be the important reasons for the significantly higher incidence of depression and anxiety in PCOS population.

Previous research has indicated that obesity is a significant predictor for persistent risk of depression when analyzing the metabolic phenotype of PCOS in relation to anxiety and depression (6), A cross-sectional study of women with PCOS identified insulin resistance as a risk factor for depression (29). However, no statistical differences were found between PCOS metabolism and anxiety and depression in this study. One possible reason is the small sample size, and another is that patients with mild depression and mild anxiety constituted the majority in our study (31.6% and 30.6%, respectively). Previous studies have primarily focused on major depression, which may explain why patients do not seek medical attention until they are aware of their illness and in need of treatment. We did not follow up whether these patients received further diagnosis and treatment, so more attention should be paid to these patients. Additionally, related studies have shown that obesity impacts the occurrence of depression, but this was not observed in our study. This could be due to the fact that patients with reproductive disorders were surveyed when seeking medical treatment, allowing for early detection, intervention, psychological counseling, and further development of their condition without a correlation between obesity and depression being established. Our study found that the risk of depression and anxiety in Chinese PCOS patients varied among different ethnic groups, with a higher incidence in Han Chinese, which may be mainly due to differences in cultural cognition (30, 31).

In this study, the PHQ-9 and GAD-7 scales were utilized to assess the levels of depression and anxiety in patients. The findings indicated that PCOS patients exhibited significantly higher levels of depression and anxiety compared to the control group, which is consistent with previous related studies (8, 32). Through our study, we found that the risk of anxiety in PCOS patients was not related to their blood metabolic indexes. The clinical significance of thyroid hormones in depression has been extensively studied, with some studies suggesting that thyroid hormone supplementation may enhance and accelerate antidepressant treatment. Additionally, certain studies have found a relationship between the concentration of free thyroid hormone and the severity of depression (33), which is consistent with the results of our study. There is a statistically significant difference between FT3 in thyroid function and depression in PCOS patients. Research has indicated that alterations in thyroid hormones play a central role in the development of depression, and a retrospective study has demonstrated that lower FT3 and FT4 values can serve as predictors for adverse clinical outcomes associated with depression (34). However, there are few studies on the interaction between thyroid hormones and depression in PCOS population. Further studies are necessary to investigate the predictive value of thyroid hormones for depression in individuals with PCOS, as well as to implement early interventions for preventing persistent depression in clinical PCOS cases.

This study conducted a questionnaire survey and data analysis on the sleep status and psychological symptoms of women with PCOS, providing valuable insights for early prevention and intervention of anxiety and depression in this population. There is a lack of research in the Chinese population regarding the impact of sleep on depression and anxiety in women with PCOS, and few studies directly compare their relationship (35, 36). Some studies have suggested that sleep-related variables may be involved in mental disorders, and sleep deprivation can significantly increase the odds of anxiety and depressive disorders (37). Through our study, it can be observed that sleep-related variables are risk factors for anxiety and depressive symptoms, especially sleep duration and daytime dysfunction have a strong correlation with anxiety and depressive symptoms (Table 7).

Our findings are generally consistent with those of previous studies. Similarly, sleep duration and daytime dysfunction significantly increase the risk of anxiety and depression in PCOS women (38), which indicates that sleep has a certain impact on the mental health of PCOS women. The differences were mainly due to heterogeneity between studies due to different assessment methods or classification criteria for anxiety and depression symptoms. It can be observed in our study that daytime dysfunction may exacerbate anxiety and depressive symptoms. The current study concludes that women with PCOS are highly susceptible to experiencing depression and anxiety, and the elevated prevalence of these psychological disorders is closely associated with their sleep patterns. Therefore, More research is required to investigate the risk factors associated with the high prevalence of anxiety and depression in patients with PCOS, in order to establish a more robust foundation for early clinical prevention and treatment, and to minimize the risk of depression and anxiety in these patients.

Our study is subject to several limitations. Primarily, it was a cross-sectional study and as such, the precise causal relationship between sleep and anxiety and depression cannot be definitively confirmed. Therefore, further prospective studies are necessary to validate these findings. Second, data on sleep variables were derived from standardized assessment scales, but most variables were derived from patient-reported questionnaires and may be subject to information bias. Third, the sample for this study was limited to women with PCOS who visited our hospital for reproductive disorders and voluntarily completed the questionnaire, which may limit the generalizability of our findings to the entire PCOS population as it only included patients attending the Second Hospital of Lanzhou University.

## 5 Conclusion

According to our study results, most of the anxiety and depression symptoms in PCOS patients are at a mild level, and the sleep status of PCOS patients is closely related to their anxiety and depression symptoms. These findings may contribute to targeted interventions to help PCOS patients prevent and improve anxiety and depression symptoms. In addition, further research is needed to explore the causal relationship between sleep and the occurrence and development of depression and anxiety in PCOS, so as to provide some guidance for clinical intervention and treatment.

## Data Availability

The raw data supporting the conclusions of this article will be made available by the authors, without undue reservation.
